# Role of social determinants of health in differential respiratory exposure and health outcomes among children

**DOI:** 10.1186/s12889-022-14964-2

**Published:** 2023-01-17

**Authors:** Jagadeesh Puvvula, Jill A. Poole, Yeongjin Gwon, Eleanor G. Rogan, Jesse E. Bell

**Affiliations:** 1 Department of Environmental, Agricultural, and Occupational Health, College of Public Health, University of Nebraska Medical Center, Omaha, NE USA; 2 Division of Allergy and Immunology, Department of Medicine, University of Nebraska Medical Center, Omaha, NE USA; 3 Department of Biostatistics, College of Public Health, University of Nebraska Medical Center, Omaha, NE USA; 4 School of Natural Resources, University of Nebraska-Lincoln, Lincoln, NE USA; 5 Daugherty Water for Food Global Institute, University of Nebraska, Lincoln, NE USA

**Keywords:** Social determinants of health, Pediatric asthma, Asthma disparities, Environmental injustice

## Abstract

**Background:**

Attributes defining the Social Determinants of Health (SDoH) are associated with disproportionate exposures to environmental hazards and differential health outcomes among communities. The dynamics between SDoH, disproportionate environmental exposures, and differential health outcomes are often specific to micro-geographic areas.

**Methods:**

This study focused on children less than 20 years of age who lived in Douglas County, Nebraska, during 2016–2019. To assess the role of SDoH in differential exposures, we evaluated the association between SDoH metrics and criteria pollutant concentrations and the association between SDoH and pediatric asthma exacerbations to quantify the role of SDoH in differential pediatric asthma outcomes. The Bayesian Poisson regression model with spatial random effects was used to evaluate associations.

**Results:**

We identified significant positive associations between the annual mean concentration of criteria pollutants (carbon monoxide, particulate matter_2.5_, nitrogen dioxide, sulfur dioxide) with race (Non-Hispanic Black and Hispanic/Latino), financial stability, and literacy. Additionally, there were significant positive associations between higher rates of pediatric asthma emergency department visits and neighborhoods with more Non-Hispanic Black children, children without health insurance coverage, and households without access to a vehicle.

**Conclusions:**

Non-Hispanic Black and Hispanic/Latino children living in Douglas County, NE experience disproportionately higher exposure to criteria pollutant concentrations. Additionally, higher rates of asthma exacerbations among Non-Hispanic Black children could be due to reduced access to respiratory care that is potentially the result of financial instability and vehicle access. These results could inform city planners and health care providers to mitigate respiratory risks among these higher at-risk populations.

**Supplementary Information:**

The online version contains supplementary material available at 10.1186/s12889-022-14964-2.

## Introduction

Asthma is a chronic disease that affects 14% of the global and 10.5% of the United States (U.S.) pediatric population [1, 2]. Environmental factors such as infectious agents, tobacco smoke, and allergens/irritants are crucial in triggering asthma exacerbations [3]. Few studies have demonstrated the role of social or structural determinants of health (SDoH) (such as ethnicity, poverty, and parental education) in disproportionate exposure to environmental hazards [4, 5]. Neighborhoods with residential segregation driven by SDoH have higher exposure to environmental hazards and poor respiratory health outcomes among children [6–8]. In the United States, marginalized communities that are influenced by social vulnerabilities (such as race/ethnicity and poverty) were associated with higher exposure to criteria pollutants [i.e., carbon monoxide (CO), sulfur dioxide (SO_2_), nitrogen dioxide (NO_2_), ozone (O_3_), and particulate matter (PM_2.5_ & PM_10_)] which were demonstrated at both community-scale [9–11] and personal monitoring based studies [12].

The role of SDoH on human health could be explained using Diderichsen’s model for the social production of diseases [13]. This model conceptualized the role of SDoH on social stratification that mediates differential exposure, vulnerability, and health consequences [13]. Institutional arrangements in the U.S. during 1930–1960 played a substantial role in residential segregation or redlining of housing areas, using social and economic forces (race, poverty, joblessness, educational failure) [14]. Due to social and economic vulnerability, certain communities were clustered in neighborhoods which the Home Owners’ Loan Corporation (HOLC) considered hazardous [15]. The majority (64%) of the neighborhoods categorized as hazardous by the HOLC during the 1960s translated into low-moderate income or ethnic minority communities over time [15]. Structural disparities that include historic redlining and lending discrimination driven by social position (race/poverty) play a crucial role in segregating disadvantaged communities and multiplying their environmental health burden [16–18].

A study that included 202 U.S. cities with HOLC maps reported a 56% higher exposure to nitrogen dioxide and 4% higher exposure to particulate matter (PM_2.5_) among individuals living in neighborhoods categorized as hazardous compared to those categorized as desirable [6]. Disproportionate exposure to air pollutants could be further translated into poor respiratory health outcomes. Nardone et al. (2020) reported that emergency visits due to asthma were 2.5 times higher in the redlined census tracts in California [19]. Similarly, Zarate et al. (2021) reported statistically significant associations between SDoH (racial, access to vehicle, and education) and a higher rate of pediatric asthma exacerbations in Travis County, Texas.

The Omaha metropolitan area in Douglas County, Nebraska, is one such area that was substantially impacted by historic redlining practices from the 1930s [20]. The redlining in Omaha led to three geographic clusters: North (predominantly Black race); south (stockyard workers); west (predominantly White race) [20]. Racial segregation in Douglas County, Nebraska, continued to exist in 2010, where the Non-Hispanic Black population is still clustered towards the north and the Hispanic/Latino population in the south [20]. Despite growing evidence on the race/ethnic disparities in Douglas County, Nebraska [21], there has been a limited focus on the disparities associated with pediatric asthma. Additionally, the existing literature either focused on the potential role of SDoH on differential environmental exposure or outcomes. This study aims to assess disproportionate exposure to criteria pollutants and differential asthma outcomes among children in Douglas County, Nebraska.

## Methods

### Study area

This study evaluated the association between 1) annual mean concentration of criteria pollutants and SDoH indicators to assess disproportionate exposures; 2) community-level SDoH indicators and pediatric asthma exacerbations to estimate differential outcomes among 32 zip-code areas of Douglas County, Nebraska. The associations were evaluated at a zip-code scale, and the results are discussed by clustering Douglas County into the Omaha metro area (northeast, southeast, northwest, and southwest) and western Douglas County (communities designated as Bennington, Ralston, and Valley) [22].

### Pediatric asthma exacerbations

We obtained the pediatric asthma exacerbations data over four years (2016–2019) from the Nebraska Hospital Association [23]. The dataset includes children (age ≤ 19 years) living in Douglas County, Nebraska, who visited an emergency room with a primary complaint of asthma exacerbation [ICD-10: J45.x; except exercise induced-asthma (J45.990)]. We then calculated the annual mean count of asthma-related emergency department visits by gender and Douglas County zip-code areas for the analysis. We obtained male and female pediatric populations by zip-code area from the 5-year American Community Survey (ACS) collected in 2019 using the US Census Application Programming Interface (API) and “tidycensus” [24, 25]. Additionally, we estimated the gender-specific average rate of pediatric asthma exacerbations per zip-code per 10,000 children.

### Social determinants of health

**Fig. 1:**
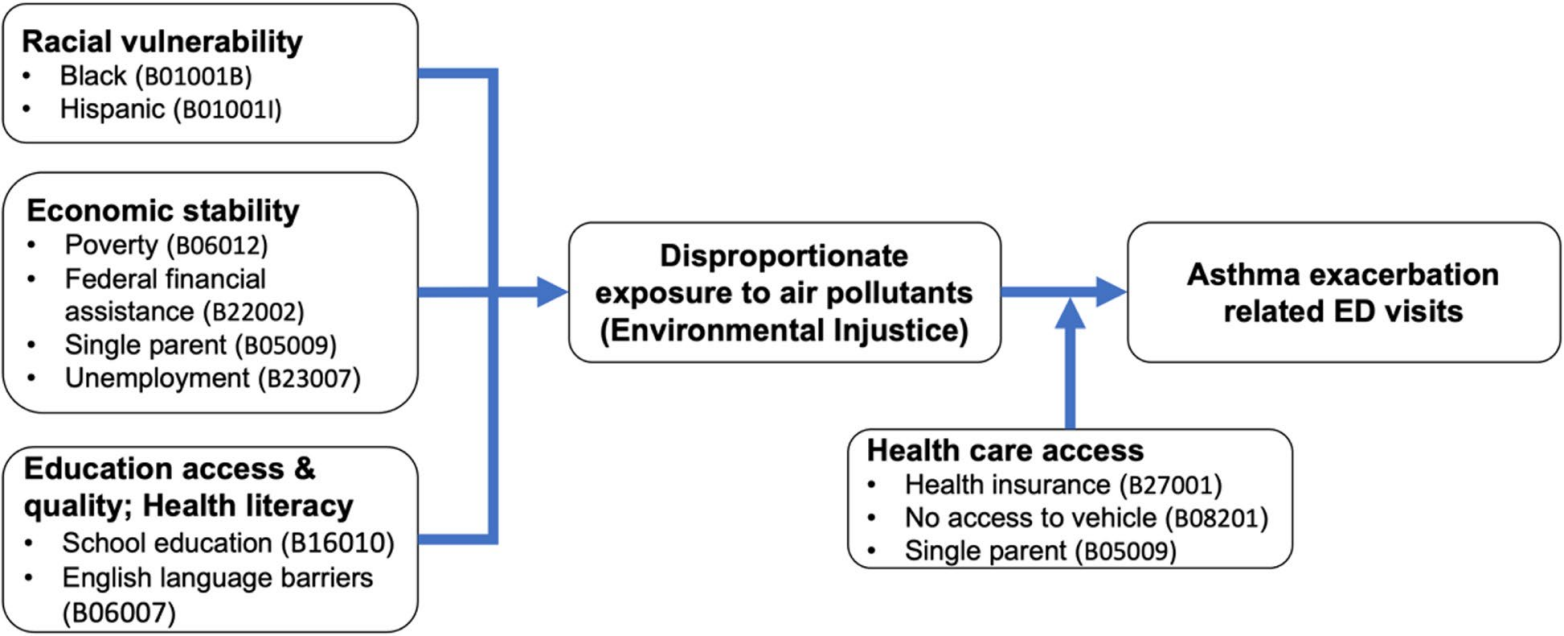
Conceptual framework exploring association between SDoH and pediatric asthma exacerbations. B01001B-Non-Hispanic Black or African American; B01001I-Hispanic or Latino; B06012-Poverty status in the past 12 months; B22002-Household with children and received Food stamps/Supplemental Nutrition Assistance Program; B23007-Households with children under 18 years and unemployed parents; B16010-Households with adults (> 25 years) and education less than high school; B06007-Individuals with limited ability to speak English; B27001-Childern without health insurance; B08201-Households without access to a vehicle. Alpha numeric codes corresponding to each variable are identifiers from the Census data

Based on the existing literature [7, 26, 27], we identified ten metrics from the 5-year (2015–2019) ACS that approximate the community scale vulnerability. The SDoH metrics were obtained using the US Census Bureau API and the “tidycensus” R-library [24, 25]. These vulnerability factors are grouped into racial vulnerability (Non-Hispanic Black, Hispanic/Latino populations), economic stability (poverty, federal assistance, single parent, unemployment), education (school education, English language barriers), and health care access (health insurance, no access to vehicle) (Fig. 1). We then transformed these variables into a percentage scale. Ethnicity and health insurance were stratified by gender and were specific to the pediatric age group. At the same time, SDoH metrics: single parent, federal financial assistance, poverty, language barrier, unemployment, education, and access to a vehicle were measured at the household scale.

### Criteria pollutants

The annual mean concentration of criteria pollutants [carbon monoxide, nitrogen dioxide, ozone, sulfur dioxide, and particulate matter (PM_2.5_ and PM_10_)] over 8 years (2007–2015) were obtained at the census tract scale from the Center for Air, Climate and Energy Solutions (CACES) database [28–31]. The annual mean criteria pollutant concentrations were obtained using the Land Use Regression (LUR) model output. We then calculated the annual mean concentration per zip-code area to match the spatial resolution of the SDoH data used in this study. The pollutant concentrations per zip-code area were then standardized using the z-score scale [32].

### Statistical analysis

The dataset contains the annual mean count/rate of pediatric asthma exacerbations, SDoH metrics, and annual mean criteria pollutant concentrations at a zip-code scale. We calculated the Spearman correlation coefficients between the rate of pediatric asthma exacerbations, SDoH metrics stratified by gender, and criteria pollutant concentrations. Further analysis was conducted considering binary structures for the zip-code spatial weights using the “nb2listw” function from the “spdep” package version 1.2–5 [33]. We then evaluated the presence of spatial clusters for the rate of asthma exacerbations, SDoH metrics, and criteria pollutants using Moran’s I test and simulated for 999 permutations to obtain the results allowing the alpha (false positive) value up to 1% (*p*-value< 0.001) [34]. Moran’s I value could have values ranging from − 1 to + 1, where − 1 indicates strong dispersion, 0 indicates randomness, and + 1 indicates strong clustering [35].

The association between mean criteria pollutant concentrations and SDoH metrics was assessed using the Bayesian Poisson regression models with *Leroux* conditional autoregressive prior [36]. We considered this statistical model to account for possible spatial autocorrelation between the zip-code areas included in this study [37]. The analysis was conducted using an intrinsic conditional autoregressive prior, to minimize the correlation between zip-code areas that do not share a boundary (non-adjacent zip-code areas) otherwise known as spatial random effect due to autocorrelation [37–39]. For area level data, adjacent spatial weights with a binary structure were utilized to assess spatial autocorrelation [40]. The statistical model was implemented using the SDoH metric as the dependent, criteria pollutant concentration z-score as an independent variable, and pediatric population by gender or households per zip-code as an offset term (Fig. 1). Similarly, the association between the pediatric asthma exacerbations and SDoH metrics was assessed using the gender-specific annual mean count of pediatric asthma-related emergency department visits per zip-code as the outcome and pediatric population per zip-code as an offset term. The analysis was conducted using R version 4.1.2.

This study was approved by the University of Nebraska Medical Center Institutional Review Board (UNMC IRB) – Protocol #0629–21-EP.

## Results

**Fig. 2:**
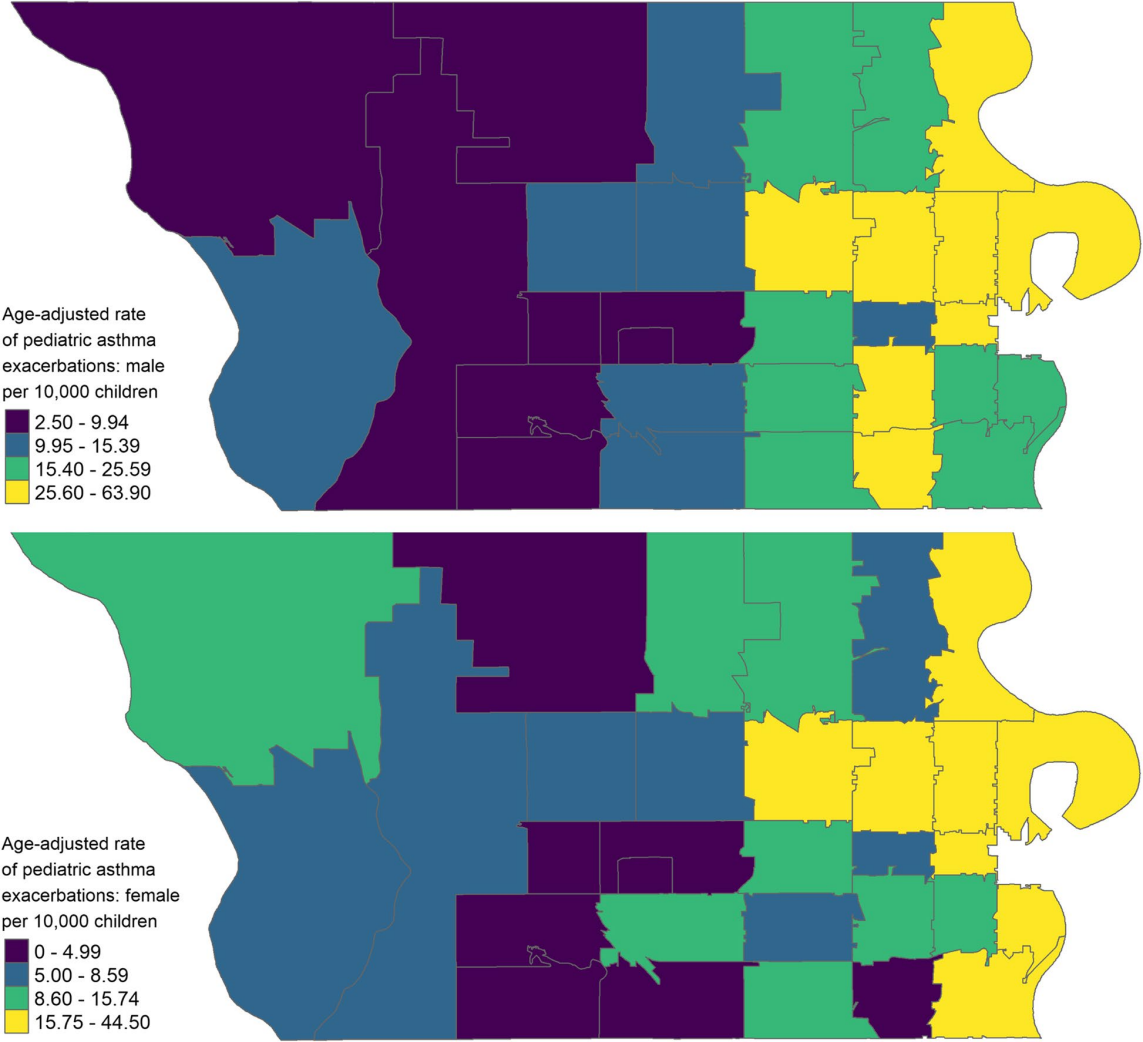
The age-adjusted rate of pediatric asthma exacerbations per 10,000 children per year per zip-code area, stratified by gender

This study included 4195 children living in Douglas County, Nebraska, who visited an emergency room due to an asthma exacerbation episode. During the study period, the annual average rate of asthma exacerbations among males ranged from 2.5–63.9 per 10,000 children per zip-code area. Similarly, the rate among females ranged from 0 to 44.5 per 10,000 children per zip-code area. We identified statistically significant clusters of pediatric asthma exacerbations in the northeastern and southeastern regions of Douglas County for both male and female children (Fig. 2, Table 1).

**Table 1:** Spatial clustering of pediatric asthma exacerbations, SDoH variables, and air pollutant concentrations

Variable	Clustered region	Moran-I	***P***-value
Asthma rate – Male	NE & SE	0.55371	0.001
Asthma rate – Female	NE & SE	0.52194	0.001
Male Non-Hispanic Black	NE	0.58897	0.001
Male Hispanic	SE	0.58831	0.001
Male health insurance	NE & SE	0.25855	0.005
Female Non-Hispanic Black	NE	0.55408	0.001
Female Hispanic	SE	0.48350	0.001
Female health insurance	NE & SE	0.47093	0.001
Single parent	NE & SE	0.47628	0.001
SSI_SNAP	NE & SE	0.00102	0.100
No vehicle	SE	0.54328	0.001
Poverty	SE	0.45598	0.001
Language	SE	0.03589	0.075
Education	NE &SE	0.22075	0.005
Employment	NE & SE	0.05499	0.146
PM2.5	NE & SE	0.57746	0.001
PM10	NW & SW	0.43162	0.001
O3	NW & SW	0.67433	0.001
SO2	NE & SE	0.50680	0.001
NO2	SE	0.66731	0.001
CO	SE	0.66907	0.001

*NE *Northeast, *SE *Southeast, *NW *Northwest, *SW *Southwest

There were statistically significant spatial clusters among 7 of the 10 SDoH metrics and the 6 criteria pollutants included in this study (Table 1, S 1, S 2-A, S 2-B). From the correlation analysis, we identified statistically significant positive correlations between the rate of male pediatric asthma exacerbations and SDoH metrics (Non-Hispanic Black population, households with children living with single parents, households with children living under federal poverty limit, and households without access to a vehicle), criteria pollutant concentrations (SO_2_, NO_2_, PM_2.5_, and CO) (S 3). The strength of correlations between the rate of pediatric asthma exacerbations and social determinants of health varied by gender. Among the female population, there were statistically significant positive correlations between the rate of asthma exacerbations and SDoH metrics (Non-Hispanic Black population, Hispanic/Latino population, children without health insurance, households with children living with single parents, households without access to a vehicle, households with children living under federal poverty limit), criteria pollutant concentration (PM_2.5_, sulfur dioxide) (S 3).

### Disproportionate exposure to air pollutants

**Fig. 3:**
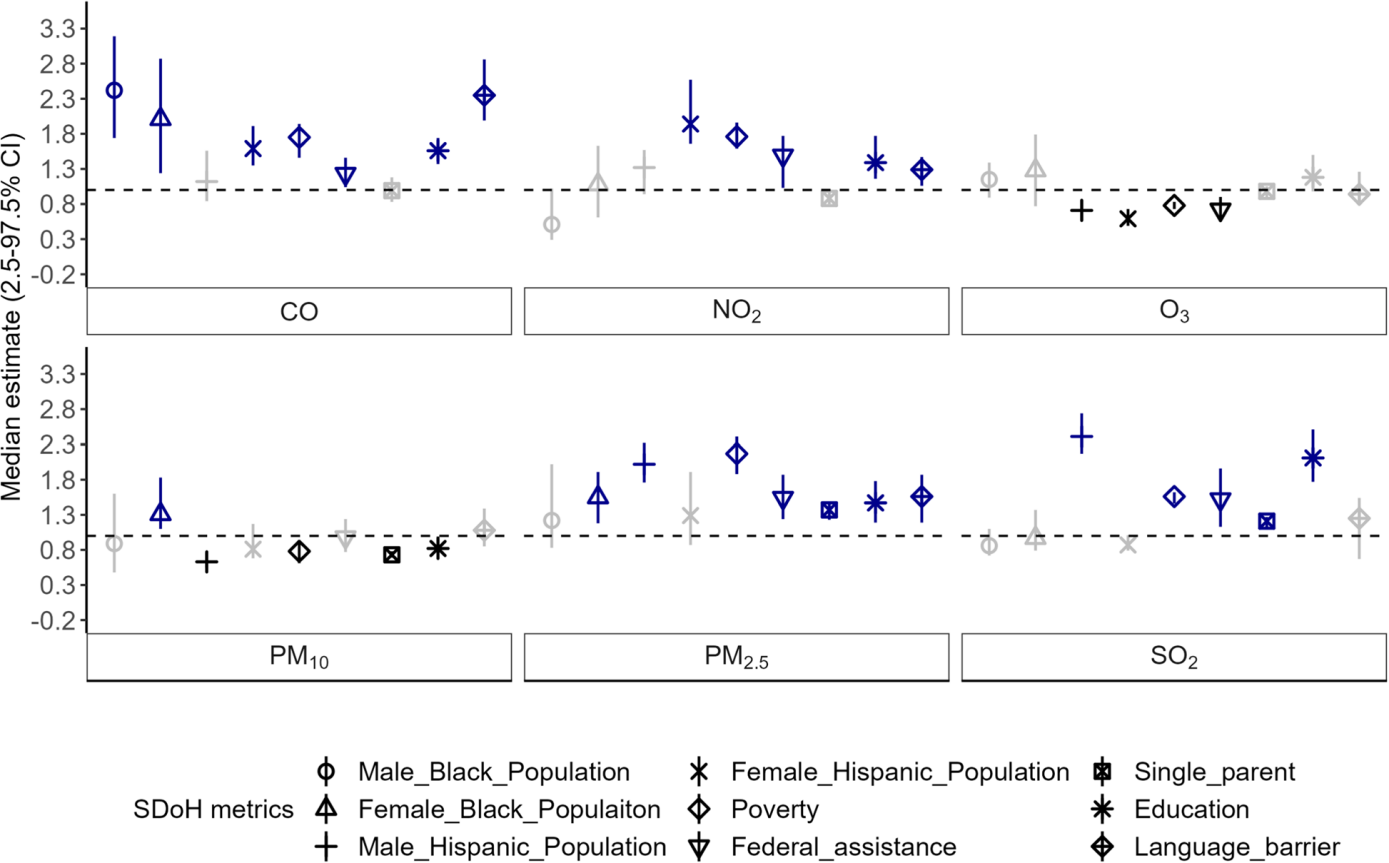
Disproportionate air pollutant exposures: Association between criteria pollutant concentration and SDoH metrics. The x-axis represents median effect estimate and corresponding 95% credible interval. The y-axis represents 9 SDoH metrics included in the analysis. Each facet in this figure correspond to 6 criteria pollutants included in this study. Effect estimates with statistically significant positive associations were highlighted in blue, statistically significant negative associations in black and statistically non-significant association in grey. Statistical significance of the effect estimates was determined based on the variables with 95% credible interval range that do not intersect 1 (dotted line)

We identified statistically significant (a credible interval that does not include zero) positive associations between SDoH metrics and criteria pollutants included in this study (Fig. 3). Every unit increase in carbon monoxide z-score is associated with up to a 2.4 fold increase in the Non-Hispanic Black population [male: 2.42, 95% credible interval (CI):1.74–3.19; female: 2.01, 95% CI:1.24–2.87], 59% increase in female Hispanic/Latino population (95% CI: 35–91%), 75% increase in households under the federal poverty level (95% CI: 46–94%), 23% increase in households received federal assistance (95% CI: 4–46%), 56% increase in adults with less than high school education (95% CI: 37–74%) and 2.35 fold increase in individuals with limitations to speaking English (95% CI: 1.99–2.86). Every unit increase in nitrogen dioxide z-score is associated with a 94% increase in the female Hispanic/Latino population (95% CI: 1.66–2.57), 76% increase in households under the federal poverty (95% CI: 59–96%), 49% increase in households received federal assistance (95% CI: 3–77%), 39% increase in individuals with education less than high school (95% CI: 16–77%) and a 29% increase in individuals with limitations to speaking English (95% CI: 6–47%). Every unit increase in sulfur dioxide z-score is associated with a 2.41-fold increase in the male Hispanic/Latino population (95% CI: 2.17–2.74), 56% increase in households below the federal poverty level (95% CI: 46–62%), 52% increase in households received federal assistance (95% CI: 13–96%), 21% increase in households with single parents (95% CI: 9–27%) and a 2.11 fold increase in individuals with education less than high school (95% CI: 1.77–2.51).

Every unit increase in particulate matter of diameter less than 10 um z-score is associated with a 31% increase in the female Non-Hispanic Black population (95% CI: 1–83%). Every unit increase in particulate matter of diameter less than 2.5 um z-score is associated with a 55% increase in the female Non-Hispanic Black population (95% CI: 18–91%), 2.02 fold increase in the male Hispanic/Latino population (95% CI: 1.76–2.32), 54% increase in households below the federal poverty limit (95% CI: 24–87%), 54% increase in households received federal assistance (95% CI: 24–87%), 37% increase in households with single parents (95% CI: 23–49%), 47% increase in individuals with education less than high school (95% CI: 19–78%) and 56% increase in individuals with limitations to speaking English (95% CI: 19–87%).

### Differential pediatric respiratory health outcomes

**Fig. 4:**
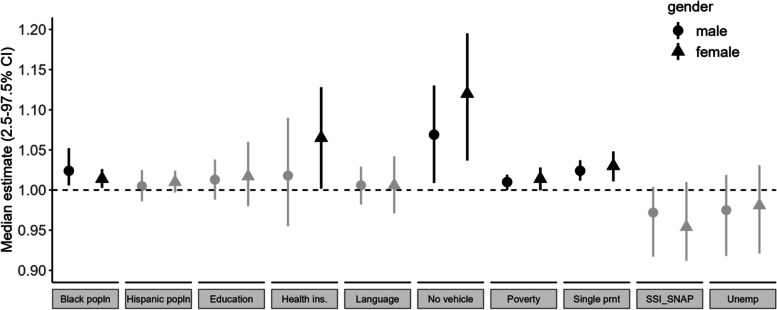
Association between social determinants of health and pediatric asthma exacerbation-related emergency department visits. The x-axis represents the median effect estimates and 2.5–97.5% credible interval; the y-axis represents 10 SDoH metrics. The effect estimates and confidence intervals were generated from the spatial autocorrelation model. The effect estimates with credible intervals that do not intersect 1 (dashed line) were considered significant and are represented using black color and non-significant associations using grey. The effect estimates corresponding to the male population were represented using circles and triangles for females

We identified statistically significant associations between the rate of asthma exacerbation-related emergency department visits and 5 of the 10 SDoH metrics included in this study (Fig. 4). Among the five SDoH metrics associated with pediatric asthma exacerbations, the metric estimating households without access to a vehicle had the highest effect estimate, followed by a single parent, Non-Hispanic Black race, and poverty. The metric of children without health insurance was associated with higher asthma exacerbations among females.

Hereafter, we refer to 10% of the SDoH metric as a unit of SDoH per zip-code area. During the study period, a unit increase in households without access to a vehicle is associated with a 6.9% (CI: 9–13%) increase in the rate of male asthma exacerbations and an 11.8% (CI: 4.0–19.4%) increase in female asthma exacerbations. A unit increase in households with children living with single parents is associated with up to a 3% increase in the rate of pediatric asthma exacerbations (male: 2.4% (CI: 1.2–3.6%); female: 3% (CI: 1.1–4.9%)). Similarly, a unit increase in Non-Hispanic Black pediatric population is associated with up to 2.5% increase in rate of pediatric asthma exacerbation related emergency visit (male: 2.5% (CI: 0.3–4.1%); female: 1.5% (CI: 0.2–2.6%)). A unit increase in households with children living under federal poverty limit is associated with up to 1.5% increase in pediatric asthma exacerbations (male: 1.1% (CI: 0.01–2.1%); female: 1.5% (CI: 0.02–3.6%)).

The association between the SDoH metric children without health insurance per zip-code area and the rate of pediatric asthma exacerbations was limited to the female population. A unit increase in children without health insurance is associated with 6.5% (CI: 0.9–13.0%) increase in the rate of asthma exacerbation-related emergencies. The SDoH indicators, such as Hispanic/Latino population, households with adults with less than secondary education, households with language barriers, households with children living with unemployed parents, and households received food stamps or federal assistance, were not associated with pediatric asthma exacerbation-related ED visits.

## Discussion

This study is focused to understand the interplay between SDoH metrics that could result differential exposure to air pollutants, and pediatric asthma exacerbations, using zip-code spatial delineations. This approach, by highlighting certain SDoH metrics and quantifying their role in pediatric asthma exacerbations, at a local scale (Douglas County, Nebraska), could potentially drive our results to action (facilitating policy change). Few studies have evaluated the potential role of SDoH metrics while evaluating the association between air pollutant exposures and health outcomes. This study is focused on the associations between SDoH and potential air pollutant exposures, along with pediatric asthma exacerbations, at a zip-code scale. By evaluating the role of SDoH on differential exposures and outcomes, we aimed to explore environmental inequities and health inequities (access to health care). Our results suggest children living in the northeastern and southeastern region of Douglas County, NE, were potentially exposed to higher levels of criteria pollutants that resulted in higher rates of pediatric asthma exacerbations. We observed disproportionate exposure to criteria pollutants among zip-code areas concentrated with Non-Hispanic Black and Hispanic/Latino children. Additionally, the observed higher rate of asthma exacerbations among Non-Hispanic Black children could result from reduced access to respiratory care potentially mediated via financial instability and vehicle access.

We observed statistically significant spatial clustering of the age-adjusted rate of pediatric asthma exacerbation-related emergency department visits in the southeast and northeastern regions of Douglas County. The spatial pattern of criteria pollutants (CO, SO_2_, NO_2_, and PM_2.5_) and SDoH metrics showed clustering that followed a similar pattern as the asthma exacerbation-related emergencies, indicating a potential role of differential exposure to criteria pollutants and disproportionate asthma-related outcomes mediated by SDoH among children. Similar to our findings, a study based on 202 U.S. cities reported disproportionate higher exposure to criteria pollutants among redlined neighborhoods [6]. Additionally, studies conducted in New York City, New York, and Travis County, Texas, identified spatial clustering of the rate of asthma exacerbations and SDoH metrics (such as race, health insurance, and poverty) [7, 41, 42]. Our results also aligned with findings from the Douglas County child and adolescent community needs assessment report [22]. The findings from this report identified poor respiratory health (current asthma and allergies) among children living in the northeastern region of Douglas County, Nebraska [22]. The report also identified that children living in the southeastern and northeastern regions were exposed to secondhand smoke at least two times more than the children living in other zip-code areas of Douglas County, Nebraska. Additionally, the needs assessment identified a higher rate of several physical and mental health conditions clustered in the northeast and southeastern parts of Douglas County. However, there have been a paucity of studies emphasizing the role of SDoH in differential exposure to air pollutants and differential asthma-related health outcomes among children [7, 26]. Unlike these other studies, our study demonstrated a striking association between community-scale criteria pollutant concentrations with SDoH metrics and SDoH metrics with pediatric asthma exacerbations.

The associations between criteria pollutants and SDoH metrics emphasize the differential exposures among vulnerable communities. The SDoH metrics representing race, economic stability, and education were significantly associated with traffic-related air pollutants (TRAP) [such as nitrogen dioxide, particulate matter (PM_2.5_)] and fossil fuel emissions [such as carbon monoxide, sulfur dioxide, and particulate matter (PM_2.5_)] [43]. The association between TRAP and SDoH metrics could be due to the state highway-75 in the northeastern region and the interstate (I)-480/I-80 in the southeastern region [44]. The combination of relatively low literacy and poverty could play a role in disproportionately higher exposure to TRAP among Non-Hispanic Black and Hispanic/Latino children. Additionally, the association between fossil fuel emissions (carbon monoxide, sulfur dioxide) and SDoH metrics could be explained by neighborhoods with lower literacy and higher poverty overlapping with a major coal and natural-gas power plant in the southeastern region of Douglas County [45]. Similarly, the interaction between economic stability and literacy could play a role in disproportionate exposure to fossil fuel emissions among Hispanic/Latino children. Our results along the same lines as the findings from a national-scale study that included 202 U.S. cities and reported that individuals living in the neighborhoods previously categorized as hazardous (grade - D) or redlined by the Home Owner’s Loan Corporation (HOLC) are predominantly low-income and ethnic minorities (Non-Hispanic Black, Hispanic/Latino, or Asians) and are exposed to disproportionately higher air pollutant concentrations [6, 26]. Considering the findings from the literature, since 2019 there were certain initiatives from the government and non-profits addressing environmental injustice among communities. In Douglas County, Nebraska, a non-profit organization has initiated efforts on improving household living conditions among individuals with higher socioeconomic vulnerability [46]. Additionally, the US Department of Justice has initiated steps against residential segregation driven by socioeconomic status, by enforcing lending laws on banks [47–50]. Systematic action towards environmental injustice could substantially minimize environmentally relevant disparities among communities.

Transitioning to the differential pediatric asthma health outcomes, we identified significant associations between SDoH metrics such as race, economic instability, access to care metrics, and pediatric asthma exacerbation-related emergency department visits. Among the SDoH metrics included in this study, we found the highest effect estimates for households without access to a vehicle. Several other studies have also identified significant associations between access to a vehicle in a household and pediatric all-cause emergency visits [51]; pediatric asthma-related emergency visits [7]. As the pediatric population relies on caregivers for medical care, access to a vehicle within a household could play an important role in pediatric disease management. Limited access to transportation among caregivers was associated with 18–51% of missed child medical appointments (unmet health care needs) and 68–87% of missed child prescriptions [52, 53]. Results from a national survey that included 71,360 children, identified that children with two or more unmet health needs are at 2.3–6.1 times higher risk of visiting an emergency room than children without these needs [54]. Children’s unmet healthcare needs are commonly associated with poverty and insurance. Children living in households below the federal poverty level and uninsured are three times more likely to have an unmet health need [55].

The association between children living in households with a single parent and children without health insurance coverage with the rate of pediatric asthma exacerbations could also be associated with access to medical care. Findings from a cohort study conducted at Cincinnati Children’s Hospital Medical Center reported that asthma-related emergency readmissions were 44% higher among children living with single parents [56]. The overall health of the children living with a single parent could be mediated via household financial resources and residential conditions [57]. Namely, Weitoft et al. found significant associations between health insurance and female asthma exacerbation-related emergency visits [57]. Uninsured children with asthma are 1.4–2.4 times more likely to visit an emergency room than children with private insurance or a public health plan [54, 58].

The association between the Non-Hispanic Black race, households without access to a vehicle, poverty, and households with single parents with pediatric asthma exacerbations could interact or mediate exposure to environmental triggers. A pooled cohort study in the U.S. that included 5809 children reported 47% higher asthma incidence among Black children than White children, 12% higher asthma incidence among children from households below the poverty level, and 14% higher asthma incidence among households with a single parent, [59]. Additionally, exposure to secondhand smoke could drive differential respiratory health outcomes among children. The exposure to secondhand smoke was two times higher among Non-Hispanic Black children than in other racial/ethnic groups [60, 61]. Similarly, exposure to secondhand tobacco smoke was 3.4 times higher among children from households below the federal poverty limit [61]. In this study, we observed clustering of racial minorities and households below the federal poverty limit in the northeastern and southeastern regions of the county, which overlapped with the findings from the needs assessment report, which showed 51% higher exposure to secondhand smoking among children living in the northeastern and southeastern region compared to the western region of the Douglas County [22].

Our study has several strengths and limitations. The limitations of this study include the criteria pollutant concentrations and SDoH characteristics measured at a community scale. Additionally, differential exposure to criteria pollutants was assessed by evaluating associations between the long-term (2007–2015) annual average of criteria pollutants and the annual average (2015–2019) estimate of SDoH metrics. The non-overlapping temporality between criteria pollutant concentrations and SDoH metrics could introduce bias. We assessed the associations between annual mean estimates of criteria pollutants and SDoH metrics to minimize potential bias. These measurements provide an approximate community scale and may vary compared to personal measurements. As we relied on approximate measurements for criteria pollutant concentrations and SDoH metrics, our results could be influenced by non-differential misclassification bias. Therefore, further studies based on SDoH metrics measured on a personal scale would provide an accurate estimate of the role of SDoH on pediatric health. Additionally, this study did not consider several environmental triggers (pollen and mold) associated with asthma exacerbations, which could introduce unmeasured confounding [62, 63]. The strength of this study is that we quantified the role of SDoH as a driving factor towards air pollutant exposures and asthma exacerbation outcome disparities among in children in a Nebraska urban area. Nonetheless, this study explored associations between the neighborhood-level criteria pollutant concentrations and SDoH metrics with pediatric asthma exacerbation-related emergency department visits in the midwestern region.

## Conclusion

We identified community-scale associations between SDoH metrics and criteria pollutant concentrations, pediatric asthma exacerbations in Douglas County, Nebraska. Our results highlighted the potential role of SDoH metrics in disproportionate exposure to air pollutants and respiratory health inequities at a zip-code scale. This information should be used by city planners and policymakers to mitigate disproportionate air pollutant exposure among socioeconomically vulnerable neighborhoods. Additionally, translating our research findings into action, implementing need-based community development programs could substantially minimize the exposure disparities among vulnerable communities. Expanding on temporary or mobile pediatric health clinics or improving transportation options to these more vulnerable areas in Douglas County, Nebraska could potentially minimize disproportionate adverse health outcomes driven by access to care issues.

## Supplementary Information

**Additional file 1.**

## Data Availability

The analytic dataset and R code generated during and/or analyzed during the current study are available at: Puvvula, J. (2022). SDoH_pasthma: Pre_publication (V1.0). Zeonodo. Available at: 10.5281/zenodo.6862366 The pediatric asthma data used in this study is not publicly available to protect patient identifiable information. The data can be requested from the Nebraska Hospital Association, Omaha, NE. Raw format of the data is available from databases mentioned below (1).The criteria pollutant data is available at the Center for Air, Climate and Energy Solutions (CACES) database. Available at: https://www.caces.us/data. (2). SDoH metrics data is available from US Census Bureau. Available at: https://api.census.gov/data.html
