# Supplementary material for: Role of social determinants of health in differential respiratory exposure and health outcomes among children
Source: BMC Public Health. 2023 Jan 17;23:119. doi: 10.1186/s12889-022-14964-2 (PMC9847182; doi:10.1186/s12889-022-14964-2)
Supplement: Supplementary file 1 — Additional file 1. [file 12889_2022_14964_MOESM1_ESM.pdf]

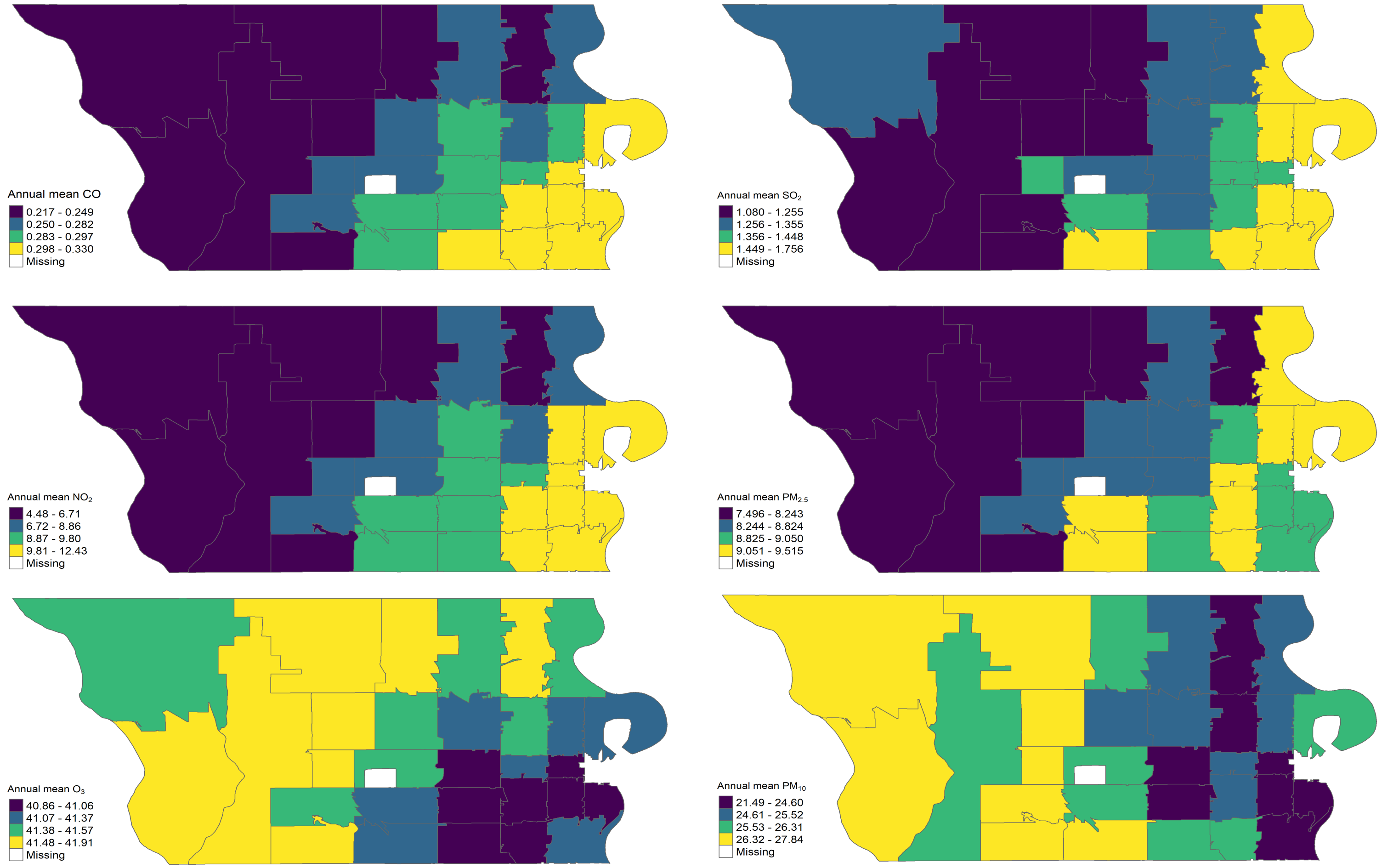

S 1. Annual mean criteria pollutant concentration per zip-code area

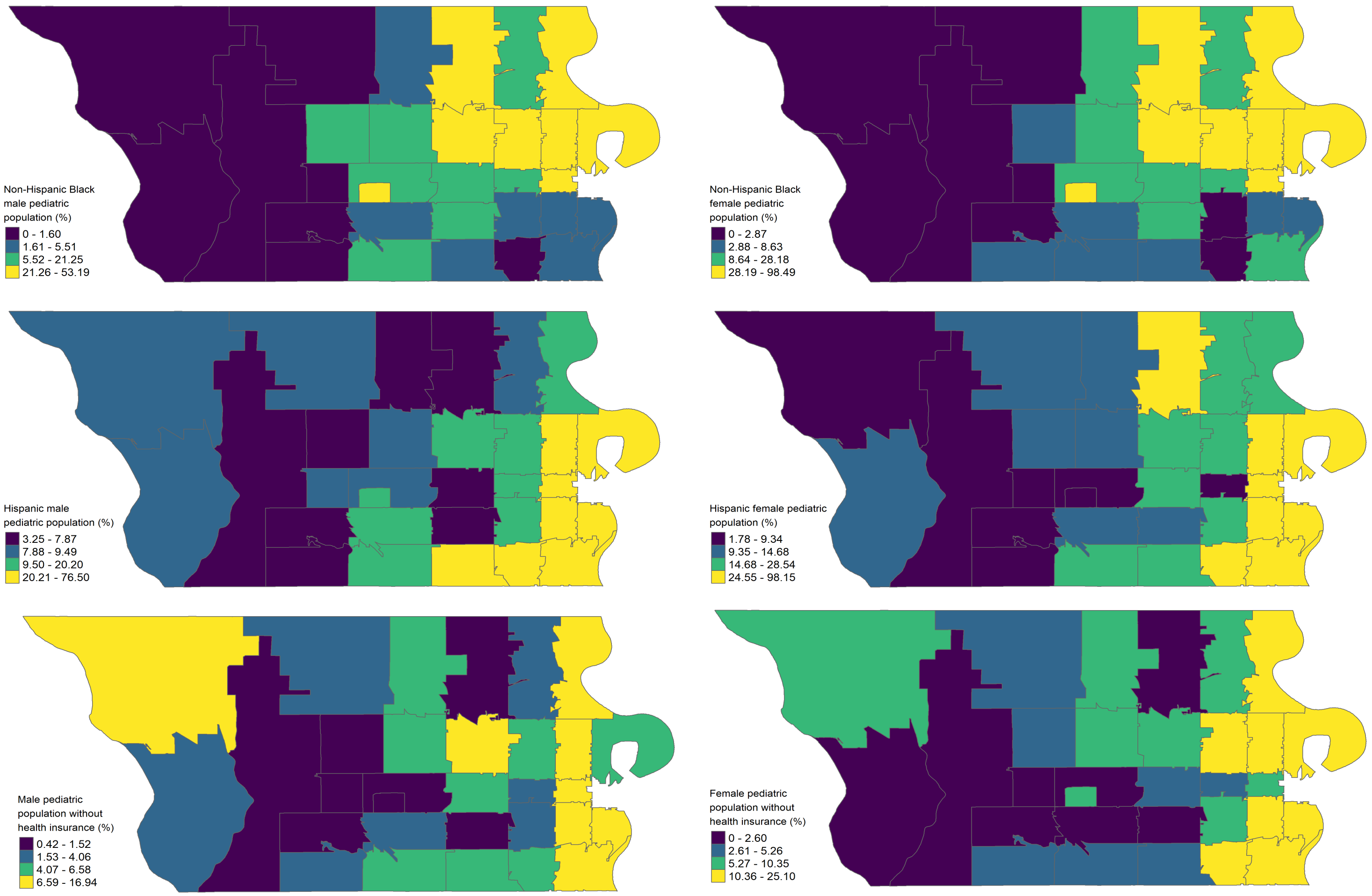

S 2-A. Spatial trend of SDoH metrics – stratified by gender

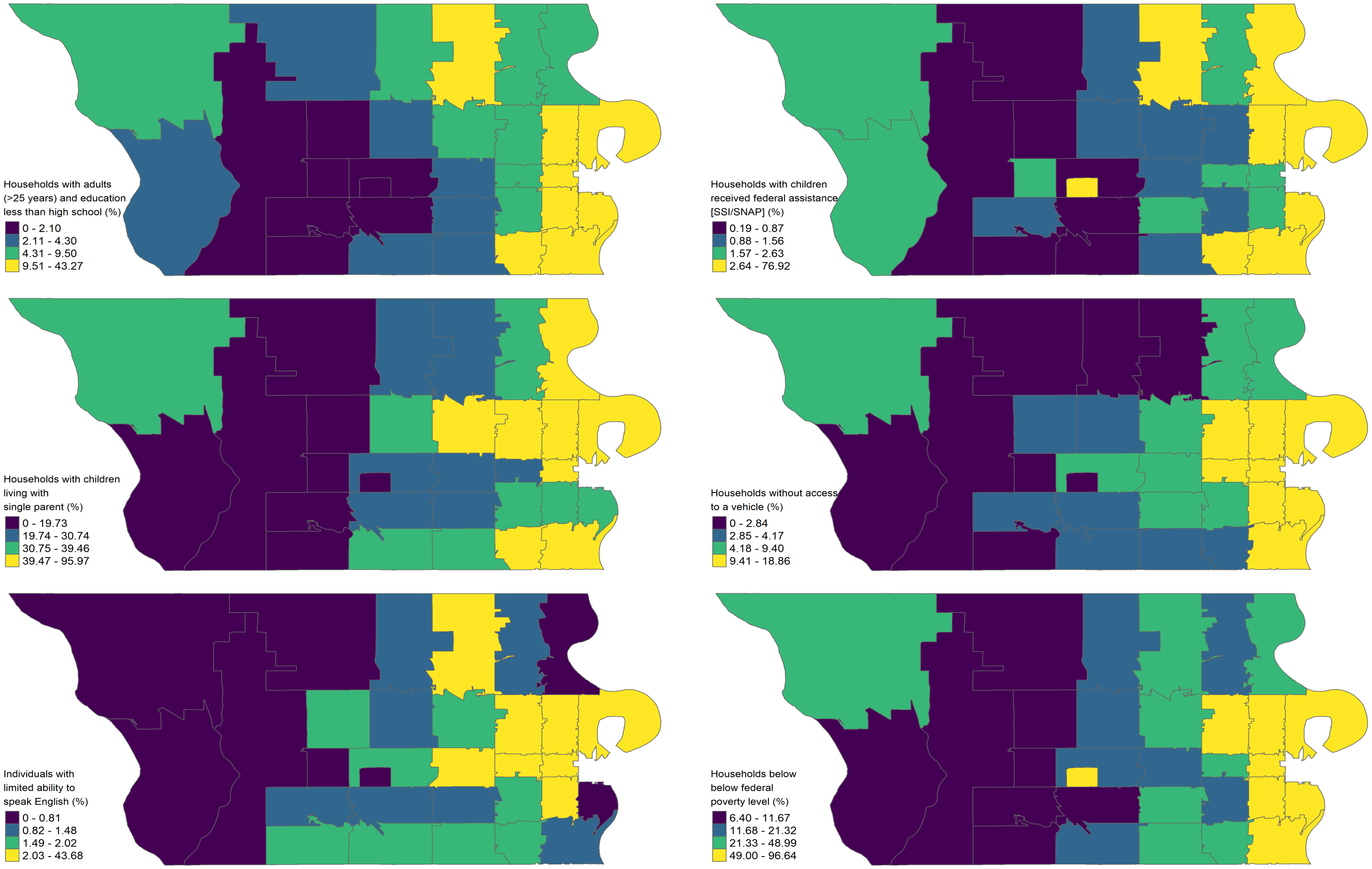

S 2-B. Spatial trend of SDoH metrics – Household scale

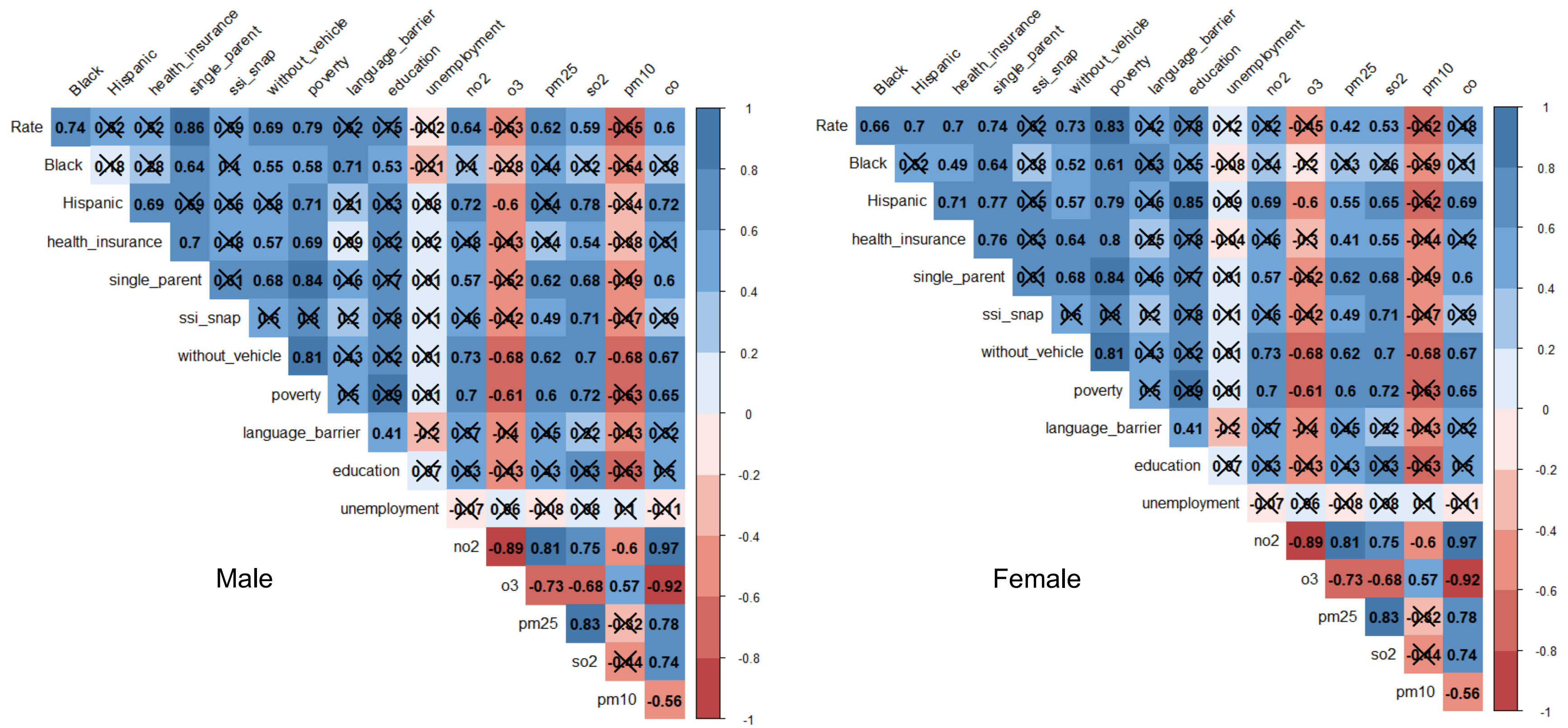

S 3. Correlation between pediatric asthma exacerbations, SDoH, and criteria pollutants stratified by gender. A- Male & B- Female. Correlation coefficients were generated using the Spearman method. Cells with statistically non-significant (p-value > 0.05) associations were stricken off.

Table S4. Summary of SDoH and asthma variables

Variable	Mean (SD)	Median (IQR)
Male asthma rate	19.2 (14.7)	15.4 (9.6-25.6)
Female asthma rate	12.3 (11.2)	8.6 (5-15.8)
Male black	13 (15.3)	5.5 (1.6-21.3)
Female black	22.1 (30.0)	8.6 (2.9-28.2)
Male Hispanic	18.2 (19.1)	9.5 (7.9-20.2)
Female Hispanic	28.1 (29.6)	14.7 (9.4-28.5)
Male without health insurance	4.5 (4.1)	4.1 (1.5-6.6)
Females without health insurance	7.4 (6.6)	5.3 (2.6-10.4)
Households with children living with single parent	32.0 (19.6)	30.8 (19.7-39.5)
Households received federal assistance (SSI/SNAP)	4.2 (13.5)	1.6 (0.9-2.7)
Households without access to a vehicle	6.8 (5.1)	4.2 (2.8-9.4)
Households under the federal poverty level	32.9 (27.9)	21.3 (11.7-49)
Households with children living with caregivers with language barriers	3.5 (7.8)	1.5 (0.8-2.0)
Households with children living with parents with education less than high school	7.3 (8.8)	4.3 (2.1-9.5)
Households with children living with parents who are unemployed	0.1 (0.1)	0 (0-0)

Rate of asthma exacerbations are estimated per 10,000 children
